# Anatomical and functional gonadotrope networks in the teleost pituitary

**DOI:** 10.1038/srep23777

**Published:** 2016-03-31

**Authors:** Matan Golan, Agnés O. Martin, Patrice Mollard, Berta Levavi-Sivan

**Affiliations:** 1 Department of Animal Sciences, The Robert H. Smith Faculty of Agriculture, Food and Environment, The Hebrew University of Jerusalem, Rehovot 76100, Israel; 2 CNRS, UMR-5203, Institut de Génomique Fonctionnelle, F-34000 Montpellier, France; 3 INSERM, U661, F-34000 Montpellier, France; 4 Universités de Montpellier 1 & 2, UMR-5203, F-34000 Montpellier, France

## Abstract

Mammalian pituitaries exhibit a high degree of intercellular coordination; this enables them to mount large-scale coordinated responses to various physiological stimuli. This type of communication has not been adequately demonstrated in teleost pituitaries, which exhibit direct hypothalamic innervation and expression of luteinizing hormone (LH) and follicle-stimulating hormone (FSH) in distinct cell types. We found that in two fish species, namely tilapia and zebrafish, LH cells exhibit close cell–cell contacts and form a continuous network throughout the gland. FSH cells were more loosely distributed but maintained some degree of cell–cell contact by virtue of cytoplasmic processes. These anatomical differences also manifest themselves at the functional level as evidenced by the effect of gap-junction uncouplers on gonadotropin release. These substances abolished the LH response to gonadotropin-releasing hormone stimulation but did not affect the FSH response to the same stimuli. Dye transfer between neighboring LH cells provides further evidence for functional coupling. The two gonadotropins were also found to be differently packaged within their corresponding cell types. Our findings highlight the evolutionary origin of pituitary cell networks and demonstrate how the different levels of cell–cell coordination within the LH and FSH cell populations are reflected in their distinct secretion patterns.

The pituitary is a master endocrine gland that integrates hypothalamic and systemic signals to produce and secrete several types of hormones; these hormones regulate a variety of physiological functions, including lactation, metabolism, reproduction and stress response^1^. Accumulating evidence from mammalian models indicates that several of the pituitary cell types are organized into complex three-dimensional networks that enable functional cell–cell coordination within homotypic cell populations^2^,^3^. Pituitary cell networks have been found to be imprinted by past experience^4^, and exhibit a high degree of plasticity as they react to feedback signals to optimize their output to the changing needs of the organism^5^,^6^,^7^. Such observations have been made for somatotropes^8^, lactotropes^4^ corticotropes and gonadotropes^9^, as well as for the non-endocrine folliculostellate (FS) cells^10^. The latter have been shown to form exceptionally long-range functional networks that have been postulated to act in the transduction of signals between distant endocrine cell populations^10^. Apart from the direct cell–cell interactions, which are largely mediated through gap junctions^11^, a complex array of paracrine signals serve to modulate pituitary cell activity, thus presenting an additional regulatory pathway in which pituitary cells interact to produce physiologically accurate output^12^.

Reproduction in vertebrates is dependent upon the coordinated actions of various hormones associated with the hypothalamus–pituitary–gonadal axis. The key modulators of reproduction are the gonadotropins (GtHs) luteinizing hormone (LH) and follicle-stimulating hormone (FSH), which are produced by the pituitary. The expression and release of GtHs from the gonadotropes is primarily regulated by the hypothalamic peptide gonadotropin-releasing hormone (GnRH) that binds to membrane receptors on the gonadotropes and triggers action potentials, a rise in cytosolic calcium, and exocytosis of GtHs into the circulation^13^,^14^. Both GtHs are glycoprotein hormones comprised of two subunits: a common α-subunit and a specific β-subunit that confers their biological specificity. In mammals and many other studied tetrapods, both GtHs are produced in the same cell but control distinct biological processes and hence require differential regulation and exhibit unique secretion patterns^15^. The differential control of LH and FSH secretion in mammals is achieved through differential packaging of the two gonadotropins^16^, differential interpretation of GnRH signaling frequency^17^,^18^, differential response to activin/inhibin signaling^19^,^20^,^21^ and a complex feedback mechanism involving gonadal steroids^15^.

All of the major components and functions of the mammalian hypothalamic–pituitary axis are largely conserved in teleost^22^; nevertheless, teleost pituitaries exhibit three traits that distinguish them from those of mammals: first, hypothalamic axons in teleosts terminate within the pituitary parenchyma^23^ whereas those of mammals discharge their output into the pituitary portal system in the median eminence, from which the signals are carried by the blood to their targets in the pituitary. Second, in contrast to the mammalian pituitary in which the endocrine cells are distributed throughout the gland, the teleost pituitary is highly compartmentalized and each cell type is located in a designated region^14^. Lactotropes and corticotropes are located in the rostral pars distalis (RPD), somatotropes, gonadotropes and thyrotropes in the proximal pars distalis (PPD), and somatolactotropes and melanotropes in the pars intermedia (PI)^14^. Third, while most studied tetrapod gonadotropes can produce LH and FSH in the same cell, fish gonadotropes secrete either FSH or LH, but not both^14^. The high level of structural and functional conservation combined with these teleost-unique traits make fish an exceptionally valuable model for studying the function and evolution of the vertebrate reproductive axis.

In fish, compartmentalization of the pituitary results in closely aggregated homotypic cell populations, suggestive of cell-cell communication. While several studies have investigated paracrine interactions in fish pituitaries^24^,^25^,^26^,^27^, very few studies have examined direct cell-cell coupling in teleosts. Preliminary works have shown gap-junction-mediated communication between unidentified pituitary cells in tilapia^28^, as well as a relatively high level of synchronization of calcium fluxes within the LH cell population^29^.

In the current study, we investigated anatomical and functional gonadotrope networks in the fish pituitary. We found anatomical contacts between fish gonadotropes and identified different degrees of coordination between LH and FSH cells, reflecting their anatomical architecture. These findings are discussed within their physiological context to provide new insights into the evolution and significance of pituitary networks in vertebrates.

## Results

**Figure 1:**
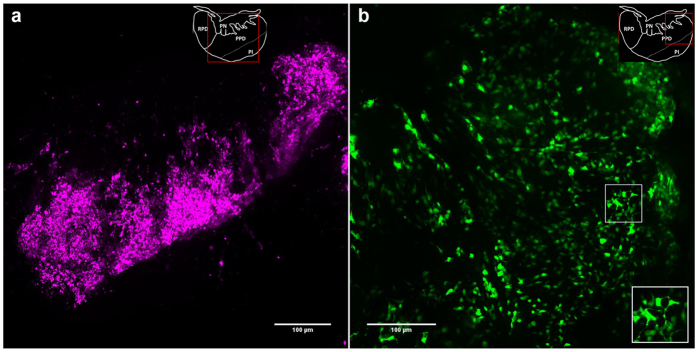
Large-scale structural gonadotrope networks in the tilapia pituitary. Imaging of transgenic tilapia pituitaries reveals that LH (**a**, LH:mCherry line) and FSH (**b**, FSH:EGFP line) gonadotropes are arranged in three-dimensional networks. LH cells form a continuous population throughout the PPD (**a**). FSH cells are distributed throughout the PPD, especially in the more dorsal area (**b**). Inset in (**b**) shows cells connected by cytoplasmic processes. A schematic drawing of the tilapia pituitary is given on the upper right of every micrograph with a red frame depicting the imaged area. RPD – rostral pars distalis; PPD – proximal pars distalis; PN – pars nervosa; PI – pars intermedia.

Using transgenic fish with labeled gonadotropes we studied cell organization in the tilapia and zebrafish pituitary. In tilapia, LH-producing cells were found to reside in tight clusters in peripheral parts of the PPD (Fig. 1a), whereas FSH-producing cells were located more dorsally, close to the dorsal projections of the pars nervosa (PN)^30^. In comparison to LH cells, FSH cells were distributed more loosely throughout the PPD. Three-dimensional imaging of transgenic fish with labeled FSH cells revealed that the contacts between these cells were far less prominent than those observed in the LH cell population (Fig. 1b). In the more ventral area of the PPD, contact between FSH gonadotropes was sometimes achieved via long cytoplasmic processes (Fig. 1b, inset).

**Figure 2:**
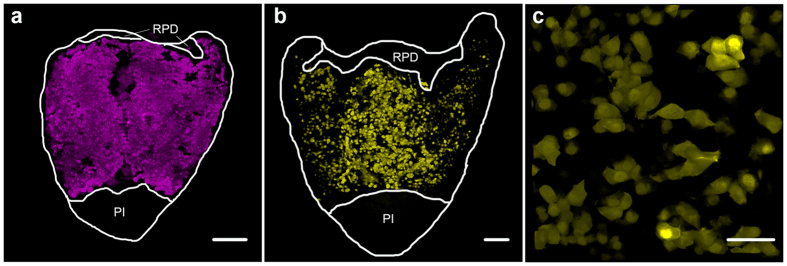
Large-scale structural gonadotrope networks in the zebrafish pituitary. Imaging of transgenic zebrafish pituitaries reveals that LH (**a**, LH:tagRFP line) and FSH (**b**,**c**, FSH:EGFP line) gonadotropes are arranged in three-dimensional networks. LH cells form a continuous population throughout the PPD (**a**). FSH cells are more loosely distributed throughout the PPD (**b**). A closer observation reveals that FSH cells form small aggregations in which cells contact each other (**c**). Both fish are adult females. Dorsal view, anterior-up, white line marks pituitary borders, PI – pars intermedia, RPD – rostral pars distalis. Bars in (**a**,**b**) −100 μm, (**c**) −20 μm.

In the zebrafish pituitary, similar trends were observed for the two gonadotrope types. Due to the small size of zebrafish pituitary we could perform imaging of the whole gland. Our transgenic models reveal that, as in tilapia, zebrafish LH cells form a continuous population throughout the gland (Fig. 2a) whereas FSH cells are more isolated from each other (Fig. 2b), although many of the membranes of FSH cells do contact neighboring cells (Fig. 2c).

**Figure 3:**
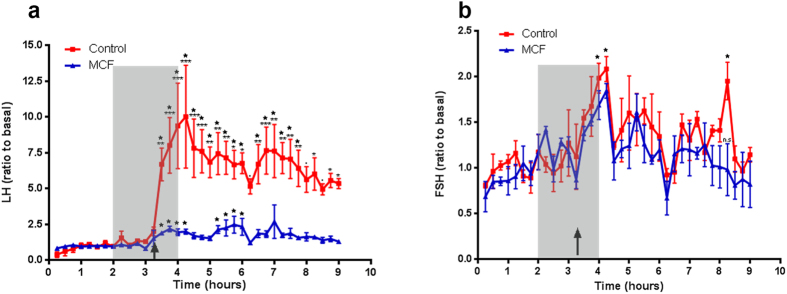
Gonadotrope release is differentially mediated by gap-junctions. Perfused tilapia pituitary fragments were stimulated with sGnRHa (arrows) with or without the presence of the gap junction uncoupler meclofenamic acid (MCF, 100 μm, shaded area). LH secretion (**a**) was significantly affected by MCF whereas FSH cell output (**b**) was not reduced by gap junction blocker application. Asterisks label the time-points that differ significantly between the two treatments (˙P < 0.05, *p < 0.01, **p < 0.001, ***p < 0.0001). Stars label the time-points that are statistically different from the basal secretion level within the same treatment.

To find out whether the anatomical contacts are also manifested at the functional level we measured gonadotropin release from tilapia pituitary fragments in the presence of gap-junction blockers. Two gap-junction uncouplers, carbenoxolone and meclofenamic acid (MCF), were used. Stimulation of pituitary fragments by 100 nM sGnRHa induced an approximately sevenfold increase in LH secretion and a twofold increase in FSH secretion by the tissue. Applying gap-junction blockers to perfused pituitary fragments almost completely abolished the response of LH cells to GnRH stimulation (Fig. 3a), whereas the FSH response remained largely unchanged (Fig. 3b). No difference was observed between the effect of MCF or that of carbenoxolone (Supplemental Figure). In control channels, LH generated a statistically significant response 15 minutes after stimulation (Fig. 3a) whereas the increase in FSH levels was significant 45 minutes after stimulation (Fig. 3b).

**Figure 4:**
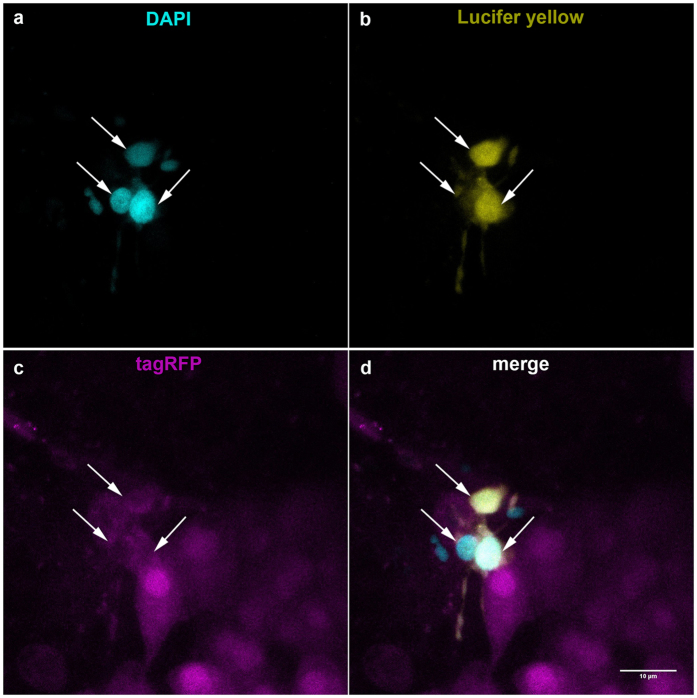
Dye coupling between neighboring LH cells. DAPI (**a**, cyan) and Lucifer yellow (**b**, yellow) were injected into a LH cell (**c**, magenta) and diffused to adjacent cells. Apart from the injected cell, two more neighboring LH cells were stained by the dyes (**d**).

To further demonstrate LH cell coupling we injected lucifer yellow (for cytoplasmatic staining) and DAPI (for nuclear staining) into identified LH cells and tested for dye transfer into neighboring cells. Over 80% (9 of 11 cells in 9 pituitaries) of LH cells showed dye coupling to at least one more LH cell (Fig. 4). The maximum number of cells labeled by the dye was 4.

**Figure 5:**
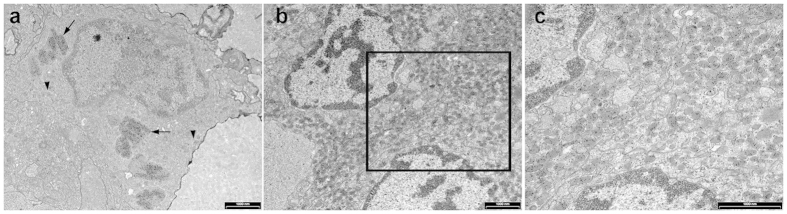
Immunogold-labeling of tilapia gonadotropes reveals distinct ultrastructure of FSH and LH cells. A typical FSH cell (**a**) exhibits large irregular bodies densely labeled with gold particles (black arrows) as well as smaller secretory granules (black arrowheads). A typical clump of LH immunogold-labeled cells (**b**,**c**) shows that the gonadotropins are packed in small oval secretory granules. A higher magnification of the boxed area in (**b**) is shown in (**c**) to provide a clearer view of the immunogold staining pattern.

Immunogold labeling with specific antibodies was used to study the ultrastructure of gonadotropes in the tilapia pituitary. At the ultrastructural level, the two studied cell types displayed distinct morphological traits. FSH cells were polygonal in shape and their cytoplasm contained unstained vesicles as well as some secretory granules. The small round granules (~150 nm in diameter) were dispersed throughout the cytoplasm and showed a low level of immunogold staining. Much stronger staining was observed in large, irregularly shaped intracellular compartments (often >1,000 nm in width) within the FSH cells (Fig. 5a). LH cells were also polygonal in shape and their cytoplasm usually contained dense oval (~200 nm on the long axis) secretory granules (Fig. 5b,c).

## Discussion

The distinct compartmentalization of the teleost pituitary, coupled with our novel transgenic lines, present a unique opportunity to study the evolution and function of cell networks within this master endocrine gland. The close association between neighboring LH cells described here in tilapia and zebrafish can also be found in other fish species such as medaka^29^, mummichog^31^, and sea bream^32^. This continuous distribution of cells throughout the gland provides the anatomical opportunity to establish gonadotrope cell networks. While the high level of compartmentalization of cell populations found in the fish pituitary has been largely lost in vertebrate evolution, the anatomical connectivity seems to have been conserved at the whole-organ level in mammals. Despite the seemingly random distribution of cells within the mammalian pituitary, three-dimensional studies in transgenic animals have revealed highly organized anatomical cell networks connecting the different types of endocrine cells in a mainly homotypic fashion^4^,^8^,^9^. The formation of these cell networks in mammals enables the pituitary to mount a robust response that is more effective by two to three orders of magnitude than the response of individual cells in primary culture^6^.

Functional coordination of gonadotrope activity has never been thoroughly studied, partly because in tetrapods, both GtHs are produced within the same cell. The segregation of FSH and LH into two distinct cell populations in fish allowed us to examine their differential organization. In classical, two-dimensional sections of fish pituitaries immunostained for FSH, the cells seem to be distributed in the PPD with little apparent connection between them^33^,^34^,^35^. However, the generation of transgenic fish with labeled FSH cells enabled 3-dimensional imaging of cell distribution and revealed that at least part of these cells maintain direct cell-cell contacts. We found that in part of the FSH cell population, the contact between neighboring cells is maintained via long cytoplasmic processes. These processes have also been observed in mammalian corticotropes and have been suggested as a means of functionally connecting cells^9^. Apart from serving as a long-distance means of communication between cells, these projections may also be utilized by the cells to contact the pituitary vascular system. This connection can increase the availability of metabolites and blood-borne stimuli to more distant cells and enable the cells to discharge their hormonal content into the circulation^36^. In fact, in larval zebrafish we found FSH cells to direct processes towards the source of GnRH stimulation^37^. In cultured pituitary cells, these processes are commonly found in gonadotropes^37^,^38^, but due to lack of the correct anatomical context, it is difficult to speculate as to their functional role. It is interesting to note that such projections do not occur in all pituitary cell types (for example, tilapia somatotropes are globular in shape and do not show cytoplasmic extensions).

To investigate whether the anatomical contacts between gonadotropes are manifested at the functional level, we applied gap-junction blockers as a means of inhibiting cell–cell communication. We found that when applied to pituitary fragments, gap-junction blockers abolished the coordinated response of LH cells but had no effect on FSH secretion, suggesting strong gap-junction-mediated coupling between LH cells. Dye-coupling of neighboring LH cells provides further evidence for the existence of gap junctions between these cells. A high level of signal synchronization was also observed in the LH cells of another teleost, medaka, while FSH cell activity was less coordinated in that species^29^. The high level of structural proximity in the LH cell clumps, revealed in both our tilapia and zebrafish models, undoubtedly serves to promote the cells’ gap-junction-mediated coupling. Our findings also suggest that if FSH cells are in fact interconnected into a functional cell network, the wiring of this network is not gap-junction-mediated. The anatomical and functional network of LH gonadotropes probably accounts for the highly coordinated secretion pattern that is the hallmark of LH secretion^14^ and the lack of such connections between FSH cells may partially explain the slower and more attenuated response of these cells to stimuli.

Studies on the hypothalamic regulation of gonadotropes in the zebrafish model provide a further explanation for the different degrees of coordination found between LH and FSH cells. In an investigation of the hypophysiotropic GnRH3 axon innervation and gonadotrope proximity to blood vessels in the zebrafish pituitary^37^, we found that only a few LH cells are contacted by GnRH3 terminals. Moreover, our observations suggested that GnRH3 signals to gonadotropes are more likely to be delivered through the blood than by direct terminal-gonadotrope contact. In contrast, individual FSH cells exhibited a closer association with the vasculature as well as with GnRH terminals than LH cells^37^. It is likely that this distinct organization is manifested functionally at the level of coordination within the two gonadotrope populations: the high level of coupling between LH cells serves to activate those cells to which hypothalamic and blood-borne signals are not available and enables the cell masses to produce the highly synchronized secretion of LH that is observed during typical LH surges. In FSH cells, on the other hand, the more direct neuronal innervation and better availability of blood-borne signals allows direct regulation of individual cells, making the coupling of neighboring FSH cells largely redundant.

Typically, in mammals LH and FSH exhibit distinct patterns of expression and secretion. In mammals, LH secretion is highly pulsatile and is characterized by sharp surges of plasma LH levels, whereas plasma FSH levels are typically more stable and less subject to rapid fluctuations^15^,^39^. This difference partly stems from the differential sorting of the β-subunits: LH is sorted into the regulated pathway, whereas FSH is sorted into the constitutive secretory pathway and its release is therefore closely associated with its synthesis^40^. While data is still lacking regarding the secretory pathways of LH and FSH in fish, our ultrastructural observations provide evidence for different regulatory pathways stemming from their distinct packaging modes. Immunogold staining using tilapia-specific antibodies revealed that LH is packed in small round or ovoid secretory granules. The cytoplasm of FSH cells, in contrast, contained only a few secretory granules that were weakly stained with gold particles, and very prominent irregular masses that were intensely stained by the gold particles. Such irregular masses have also been observed in the cytoplasm of catfish gonadotropes^41^, as well as in goldfish gonadotropes, especially after administration of estradiol^42^. Since no discrimination was made between LH- and FSH-producing cells in catfish and goldfish, it is difficult to associate this trait with a distinct type of gonadotrope. Mammalian GtHs, which are produced within the same cell, are also differentially packaged. In the mammalian gonadotrope, large globules contain FSH and, to a lesser extent, LH, whereas small globules contain only LH^43^,^44^. Granins, which are regulated secretory proteins, also differ between the two granule types: large globules usually contain chromogranin A, whereas the small secretory granules contain secretogranin II^45^. Those granules that contain LH and secretogranin II are highly affected by the regulated secretory pathway, whereas FSH secretion is granin-independent^46^,^47^. The duality in the association of FSH and LH with specific granins may explain the differential secretory patterns of these two hormones. In fish the only studied granin is secretogranin II and in particular, its derivative secretoneurin^48^. This short peptide was found to induce LH secretion from goldfish pituitaries but is not expressed in LH gonadotropes, but rather in prolactin cells^49^ as well as in oxytocin fibers that project to the pituitary^50^. It is unclear which of the two sources (hypothalamus or pituitary) is responsible for the effect on the gonadotropes^48^. In the zebrafish pituitary secretogranin III, secretogranin II and secretogranin V are among the most highly expressed non-hormonal proteins and are upregulated during sexual maturity along with an increase in LH expression^51^. While data is still lacking regarding the specific association of granins with gonadotropes in fish, our finding of differential packaging of LH and FSH suggests differentially secretory pathways for LH and FSH. Since accurate assays for measuring FSH levels in fish have been developed for only a handful of species, much less data exist on the specific plasma GtH profiles in fish. However, published reports in trout^52^ and tilapia^53^,^54^ indicate that, upon GnRH stimulation, the increase in plasma LH levels is substantially more pronounced than the increase in plasma FSH. These findings portray the FSH-secretion pattern in fish as more constitutive and imply that, as in mammals, the relative role of GnRH on FSH release is less prominent than its stimulatory effect on LH secretion.

In summary, our data suggest that LH and FSH cells in fish differ in their degree of homotypic coupling: while LH cells form a prominent structural and functional network, FSH cells appear to function more independently. This difference, along with the differential packaging of the two gonadotropins underlies their distinct release patterns. Together, these findings further enhance our understanding of the factors that contribute to the differential release of LH and FSH in fish.

## Methods

### Fish husbandry and transgenic lines

Nile tilapia (*Oreochromis niloticus*, Lake Manzala strain) were kept and bred in a recirculating water system at 26–28 °C and fed twice daily with commercial pellets (47% protein, 6% fat, Raanan Shivuk, Israel). Eggs were stripped from ovulating females 10 hours after first light and fertilized *in vitro* with freshly-stripped sperm. For transgenesis, DNA and transposase mRNA were injected into the animal pole of fertilized one-cell staged eggs. All constructs were generated using the Gateway technology and the tol2kit. Transgenesis was preformed using transposon-induced genome integration. Detailed procedures for the generation and validation of the transgenic tilapia and zebrafish lines were previously described^30^,^55^. Zebrafish were maintained and bred according to standard protocols. Details regarding zebrafish husbandry and transgenic line generation can be found elsewhere^55^. The FSH:GFP zebrafish line used in this study is hjr1(Tg(Oni.Fshb:EGFP, myl7:EGFP)). An improvement of the LH:mCherry line is described in Supplemental methods.

All experimental procedures were in compliance with the Animal Care and Use Guidelines of the Hebrew University and adhered to the National Research Council’s Guide for Care and Use of Laboratory Animals.

### Perfusion of pituitary fragments

Perfusion experiments were performed as described by Levavi-Sivan *et al.*^56^. For each channel, three pituitaries were used and each treatment was repeated in four channels (mixed-sex mature non-transgenic tilapia, gonadal somatic index 0.51 ± 0.2% and 4.32 ± 0.6% for males and females, respectively). For GtH stimulation, salmon GnRH analog [(D-Ala^6^, Pro^9^-Net)- GnRH; sGnRHa; Bachem, Inc., Torrance, CA] was administered at 100 nM for 5 min. The gap-junction inhibitors carbenoxolone or meclofenamic acid (Sigma-Aldrich, Israel) were administered at 100 μM for 1 h before, during and 1 h after GnRH stimulation. FSH and LH levels were determined in medium fractions by ELISA. Experiments were repeated three times to ensure consistency of the results.

### Antibodies

For enzyme-linked immunosorbent assay (ELISA), and immunogold labeling, we used specific antibodies raised in rabbit against tilapia FSH^33^ and LH^57^.

### ELISAs for tilapia GtHs

For the analysis of both GtHs’ levels, we used specific homologous ELISAs developed in our laboratory^58^ and validated previously^59^. Sensitivity for the measurements was 2.43 ng/ml and 1.52 ng/ml for LH and FSH, respectively. Interassay coefficient of variation was 14.8% and 12.5%, whereas intra-assay coefficient of variation was 7.2% and 8.0% for LH and FSH, respectively.

### Dye transfer assay

To visualize functional coupling between LH cells the fluorescent dyes LY (1 mM) and DAPI (1 μg/ml) were introduced into LH cells using sharp microelectrodes as described previously^60^. Pituitaries from adult female LH:tagRFP zebrafish (n = 9) were carefully excised in artificial cerebrospinal fluid (aCSF) (118 mM NaCl, 3 mM KCl, 10 mM HEPES, 25 mM NaHCO3, 2.4 mM MgCl_2_, 2.5 mM CaCl_2_ and 10 mM glucose) and mounted onto poly-l-lysine coated glass coverslips. During the injection procedure pituitaries were continuously irrigated in aCSF bubbled with 95% oxygen and 5% CO_2_. Only tagRFP-expressing cells were targeted for injection. Following injection, dye diffusion into neighboring cells was monitored visually. Pituitaries were subsequently fixed in 4% PFA and imaged under a confocal microscope.

### Transmission electron microscopy (TEM)

Pituitaries from two adult male tilapia (GSI 0.38 ± 0.05%) were processed for TEM. Tissue preparation and post-embedding immunocytochemical staining is described in detail in the Supplemental Methods section.

### Statistics

The results are presented as mean ± SEM. All statistical analysis was performed using the Prizm4 software (GraphPad, La Jolla, CA). In order to determine whether the treatment in perfusion experiments significantly affected gonadotropin secretion, perfusion results were subjected to statistical analysis by mixed effects model of response on FSH or LH versus Time using Treatment (control/MCF) as fixed factors and individual channels as random factors, with ANOVA to test the overall effect of Treatment on LH and FSH followed by Schaffer post-hoc tests. In order to test whether the GnRH stimulation significantly increased gonadotropin release from perfused pituitaries in the different treatments we compared each time point to the basal level of its specific channel using a non-parametric repeated measurement ANOVA analysis. Means are considered significant if p < 0.05.

## Additional Information

**How to cite this article**: Golan, M. *et al.* Anatomical and functional gonadotrope networks in the teleost pituitary. *Sci. Rep.* **6**, 23777; doi: 10.1038/srep23777 (2016).

## Supplementary Material

Supplementary Information
