# Supplementary material for: Anatomical and functional gonadotrope networks in the teleost pituitary
Source: Sci Rep. 2016 Mar 31;6:23777. doi: 10.1038/srep23777 (PMC4815020; doi:10.1038/srep23777)
Supplement: Supplementary Information [file srep23777-s1.pdf]

Anatomical and functional gonadotrope networks in the teleost pituitary

Matan Golan[1,2], Agnés O. Martin[2,3,4], Patrice Mollard[2,3,4] and Berta Levavi-Sivan[1]*

Supplemental Methods and Figure

Generation of LH:tagRFP zebrafish

In the current study we used an improved version of the transgenic line hjr2Tg (27) that exhibits red-labeling of LH gonadotropes. To achieve this we substituted the mCherry cassette with a tagRFP-CAAX cassette that directs the fluorescent protein to the cell membranes. The use of tagRFP eliminates the aggregation problems associated with mCherry and results in a more uniform labeling of the cells. In addition, the construct incorporates a red heart marker (cmlc2:mCherry) that facilitates the differentiation from FSH-labeled fish (green heart marker) and identification of double-labeled individuals during the embryo screening process. This new line was assigned the name hjrTg3.

Transmission Electron Microscopy

Fresh pituitaries from adult tilapia were fixed in 4% paraformaldehyde, 0.1% glutaraldeyde in 0.1 M cacodylate buffer (pH 7.4) for 2.5 h at room temperature. The tissues were then rinsed four times in cacodylate buffer and post-fixed and stained with 1% osmium tetroxide, 1.5% potassium ferricyanide in 0.1 M cacodylate buffer for 1 h. Tissues were then washed four times in cacodylate buffer, and dehydrated in increasing concentrations of ethanol, followed by 100% anhydrous ethanol and propylene oxide. Dehydrated tissues were infiltrated with increasing concentrations of agar 100 resin in propylene oxide, consisting of 25, 50, 75, and 100% resin for 16 h each step. The tissues were then embedded in fresh resin and allowed to polymerize at 60oC for 48 h. Embedded tissues were sectioned with a diamond knife on an LKB 3 microtome and ultrathin sections (80 nm) were collected onto 300-mesh nickel grids.

The post-embedding immunocytochemical procedure was generally performed according to Castel et al. (61). Grids were incubated in drops of reagent on parafilm in a wet chamber and the various steps alternated with washes in drops of Tris-buffered saline (TBS; 20 mM Tris-base, 0.9% NaCl, 0.5% BSA, 0.5% Tween-20 and 0.13% NaN3, pH 8.2). Sections were etched for 15 min in saturated aqueous sodium metaperiodate solution, followed by 60 s immersion in 1% sodium borohydride, washed five times in TBS and blocked for 1 h in 5% normal goat serum in TBS. Sections were then exposed to the primary antibodies for 1.5 h at room temperature and incubated at 4oC for 16 h. Antibody dilutions were 1:200 for LH and GH and 1:80 for FSH. For immunogold labeling, we used a secondary 12 nm colloidal gold-conjugated goat anti-rabbit IgG (Jackson ImmunoResearch Laboratories, West Grove, PA). Incubation for 1.5 h with the secondary antibody was followed by 2 min fixation in 2% glutaraldehyde and contrasting in saturated aqueous uranyl acetate and lead citrate before air-drying. Grids were viewed with a Tecnai 12 TEM 100kV (Philips, Eindhoven, the Netherlands) equipped with a MegaView II CCD camera and Analysis® version 3.0 software (SoftImaging

System GmbH, Münstar, Germany). No labeling was found on sections incubated without the primary antibodies. In stained sections, background staining was negligible (on the adjacent resin). Moreover, staining patterns were highly cell-specific and only a single cell type was labeled by each antibody, as could be judged from ultrastructural appearances. Stained FSH cells were loosely distributed whereas stained LH cells were found in dense clusters.

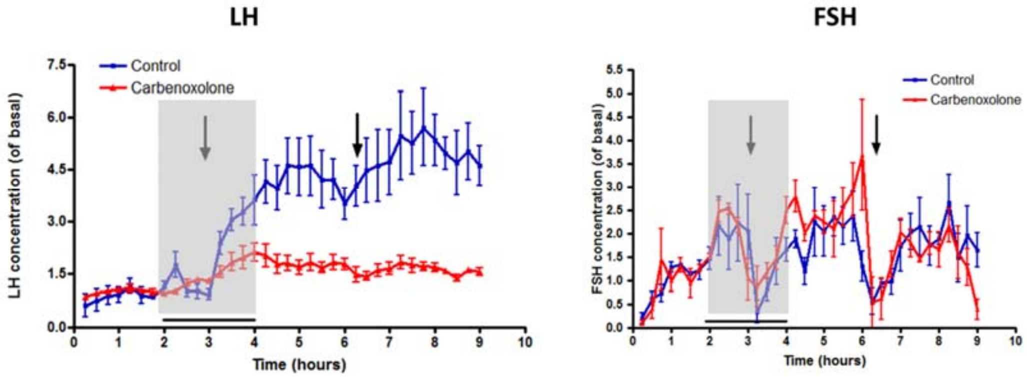

Supplemental Figure 1. Gonadotrope output is differentially mediated by gap-junctions. Perfused pituitary fragments were stimulated with sGnRHa (arrows) with or without the presence of carbenoxolone (100μm, shaded area). LH secretion (A) was significantly affected by the carbenoxolone whereas FSH cell output (B) was not reduced by gap junction blocker application.
